# First molecular detection of *Babesia vulpes* and *Babesia capreoli* in wild boars from southern Italy

**DOI:** 10.3389/fvets.2023.1201476

**Published:** 2023-08-07

**Authors:** Giovanni Sgroi, Nicola D’Alessio, Clementina Auriemma, Harold Salant, Amalia Gallo, Marita Georgia Riccardi, Flora Alfano, Simona Rea, Stefano Scarcelli, Martina Ottaviano, Claudio De Martinis, Giovanna Fusco, Maria Gabriella Lucibelli, Vincenzo Veneziano

**Affiliations:** ^1^Department of Animal Health, Experimental Zooprophylactic Institute of Southern Italy, Portici, Italy; ^2^Osservatorio Faunistico Venatorio—Campania Region, Naples, Italy; ^3^Koret School of Veterinary Medicine, Hebrew University of Jerusalem, Rehovot, Israel; ^4^Department of Veterinary Medicine and Animal Productions, University of Naples Federico II, Naples, Italy

**Keywords:** *Babesia capreoli*, *Babesia vulpes*, Italy, public health, wild boar

## Abstract

**Introduction:**

Following the increase of wild boar (*Sus scrofa*) populations in Europe, a potential risk of emerging infections by vector-borne pathogens may occur. Despite this, the circulation of piroplasmid species in these ungulates is still a neglected topic, particularly in the Mediterranean basin. Therefore, this study aimed to investigate the presence of *Babesia*/*Theileria* spp. in wild boars from southern Italy to assess the epidemiological role of these ungulates in the circulation of piroplasmids.

**Methods:**

By using a *citizen science* approach among hunters and veterinarians, wild boar spleen samples were collected in the Campania region (southern Italy) between 2016 and 2022. A combined semi-nested PCR/sequencing analysis targeting the V4 hyper-variable region of *18S* rRNA was run to detect *Babesia*/*Theileria* spp. DNA.

**Results:**

Out of 243 boars, 15 (i.e., 6.2, 95% CI: 3.4–9.9) tested positive to *Babesia*/*Theileria* spp., *Babesia vulpes* (*n* = 13, 5.3, 95% CI: 3.1–8.9) the most prevalent, followed by *Babesia capreoli* (*n* = 2, 0.8, 95% CI: 0.2–2.9). Three different *B. vulpes* sequence types were identified (i.e., ST1, ST2, ST3), with the most representative as ST1 (60%), and a single *B. capreoli* sequence type. No statistically significant difference (*p* > 0.05) were found between the presence of the pathogens and boar age, sex, province and sample collection year.

**Discussion:**

Data demonstrate for the first time the occurrence of *B. vulpes* and *B. capreoli* in wild boars, which may play a role in the biological cycle of piroplasmids. We emphasize the importance of monitoring these ungulates to prevent potential foci of *infection*. The engagement of hunters in epidemiological scientifically based surveys can constitute a technically sound control strategy of piroplasmids in a One Health perspective.

## 1. Introduction

Piroplasmids of the genus *Babesia* and *Theileria* (Aconoidasida, Piroplasmida) are global emerging tick-borne apicomplexan protozoa infecting multiple wild species, as well as domestic animals and humans (1, 2). Among more than 100 different species identified so far, some of these intracellular parasites display a high host specificity in wild mammals (3). For instance, the role of some wildlife species has been ascertained in the maintenance of certain *Babesia* spp., such as red foxes (*Vulpes vulpes*) for *Babesia vulpes*, red deer (*Cervus elaphus*) for the zoonotic *Babesia divergens* and roe deer (*Capreolus capreolus*) for *Babesia capreoli* and the zoonotic *Babesia venatorum* (3–6). Despite this, piroplasmid surveillance in wild boar (*Sus scrofa*) populations is a neglected topic due to their apparent absence in this ungulate in Europe (7). The only two *Babesia* spp. infecting boars, also common in pigs, *Babesia trautmanni* and *Babesia perroncitoi*, have been detected mostly in the 1990s via morphology without any molecular confirmation (7). The unique cases of molecular detection of piroplasmids in boars are to date reported as unspecified *Theileria* spp. in Italy (*n* = 3 out of 117) (8) and Portugal (*n* = 3 out of 65) (9), *Babesia bigemina* in Italy (*n* = 2 out of 257) (10) and a single finding of *B. divergens* out of 550 in the Czech Republic (7). This negligible occurrence of piroplasmids in boars is likely due to a low prevalence and parasitaemia and low number of tested animals (i.e., *<*100 in several epidemiological surveys) (11–13), despite the use of highly sensitive qPCR/conventional PCR protocols (7, 14). However, the role of boars in the epidemiology of piroplasmids in Europe cannot be ruled out considering that their high density (15), territorial expansion (16) and spatial overlap with other wildlife populations may increase the chance of tick infestation and piroplasmid transmission (8). Indeed, a considerable risk for new foci of emerging *Babesia* and *Theileria* infections is now evident in Europe, especially in the south and in the Mediterranean basin where great diversity of piroplasmid species (17) and high biodiversity of ixodid ticks occur (18). Some areas in these regions are also associated with a great vocation for outdoor recreational activities exposing to the risk of piroplasmid infection, as demonstrated by the high seroprevalence in hunting dogs from rural areas of southern Italy (19), where wildlife, ticks and related pathogens overlap (20). Therefore, this study aimed to investigate the occurrence of *Babesia/Theileria* spp. in wild boars from southern Italy and to assess the epidemiological role of these ungulates in the circulation of piroplasmids.

## 2. Materials and methods

### 2.1. Study area and sampling

The study was run in the Campania region, southern Italy, characterized by a typical Mediterranean temperate climate and progressively continental features of mainland and mountainous landscapes. Under the frame of a surveillance plan of wildlife by the Italian Ministry of Health (authorization no. IZSME RC 05/16), spleens of wild boars were collected from October 2016 to December 2022. Field activities were carried out in collaboration with “trained persons” (i.e., regular boar hunters educated specifically on hunting hygiene, health and food safety through specific theorical and practical courses, according to Reg. EU 853/2004) (21). Hunters culled boars and collected spleens and information (age, sex, geographic origin) under supervision of veterinarians affiliated with the University of Naples Federico II and regional health systems. In order to minimize the risk of cross-contamination, whole spleens were collected and stored at ±4°C in separate plastic biohazard bags and delivered to the necropsy room of the Department of Animal Health, Experimental Zooprophylactic Institute of southern Italy (Portici, Italy). Each spleen was flamed on the surface before sampling an aliquot from the inner portion for DNA extraction. Classes related to boar age (i.e., piglet <1 years old, juvenile 1–2 years old, adult >2 years old) were estimated by the examination of the teeth (i.e., primary and permanent teeth eruption times and root hole diameter of incisors), according to Massei and Toso (22).

### 2.2. Sample size calculation

A minimum sample size of 243 wild boars was estimated using the opensource software OpenEpi (23), inserting the following data: a population size of 84,000 boars (data supplied by the regional emergency plan of wild boars in Campania region); expected prevalence of *Babesia*/*Theileria* spp. infection in the population of 5% ±3 (i.e., 2%–8%), according to Zanet et al. (10); confidence limits of 5% and desired absolute precision of 3%.

### 2.3. DNA extraction, PCR protocol, and sequencing

One gram of spleen was individually homogenized by tissue lysis (Qiagen) in sterile PBS buffer with two 4.8 mm glass beads (Diatech Lab Line, Salerno, Italy). Each DNA extraction session included a negative extraction control (represented by an equal volume of RNase/DNase free water instead of DNA extraction elute). From 200 μL of homogenized sample, extraction of nucleic acid was obtained using a commercial kit (QIAampDNA Blood & Tissue; Qiagen, Hilden, Germany), according to the manufacturer’s instructions. A semi-nested PCR protocol targeting the V4 hyper-variable region of the *18S* ribosomal RNA gene was used for the direct detection of *Babesia*/*Theileria* spp. DNA (10). In the first round, primers RLB-F2 (5’-GACACAGGGAGGTAGTGACAAG-3′) and RLB-R2 (5’-CTAAGAATTTCACCTCTGACAGT-3′) were used in a final reaction volume of 25 μL, using Promega PCR Master Mix (Promega Corporation, WI, United States), 20pM of each primer, and ≈100 ng of DNA template measured with the Biofhotometer plus (Eppendorf, Hamburg, Germany), according to the manufacturer’s instructions. The thermocycling conditions included initial denaturation for 5 min at 95°C, followed by 25 cycles of denaturation for 30s at 95°C, 45 s annealing at 50°C and 90s extension at 72°C and a final extension of 10 min at 72°C. Amplicons (1 μL) of the first PCR round were used as template in the second round with the same primer RLB-R2 plus RLB-FINT (5’-GACAAGAAATAACAATACRGGGC-3′). The reaction mix and cycling conditions were identical in first and second rounds, except for the total number of cycles (i.e., 40) and annealing temperature (55°C) in the second round. In all PCR runs, positive (i.e., *Babesia canis* DNA of fox spleen from Italy) and negative (reaction mix plus sterile water) controls were used. All PCR products were examined on 2% agarose gels stained with GelRed (VWR International PBI, Milan, Italy) and visualized on a GelLogic 100 gel documentation system (Kodak, New York, United States). Amplicons were purified by the QIAquick PCR Purification kit (Qiagen, Hilden, Germany) and sequenced in both directions using the same primers of the second round by the BigDye Terminator v.3.1 chemistry in a 3130 Genetic Analyzer (Applied Biosystems, Foster City, CA, United States). Consensus sequences were obtained by the Geneious software version 9.0 (Biomatters Ltd., Auckland, New Zealand) (24) and compared with those available in the GenBank database by the Basic Local Alignment Search Tool (BLAST; http://blast.ncbi.nlm.nih.gov*/*Blast.cgi).

### 2.4. Statistical analysis

An exact binomial 95% confidence interval (95% CI) was established for the proportions of infection found herein. The Chi-squared or Fisherˈs exact test were used, depending on the population size, to assess any statistical differences of infection by animal age, sex, province of origin and sample collection year, while odds ratio was used for the infection risk by sex. A value of *p* < 0.05 was considered statistically significant. Statistical analyses were performed by using the online software Epitools—Epidemiological Calculators (25). The distribution of *Babesia*-positive wild boars according to provincial borders of the study area was determined using aerial imagery from Bing aerial maps software (Microsoft, Redmond, Washington, United States).

## 3. Results

**Figure 1:**
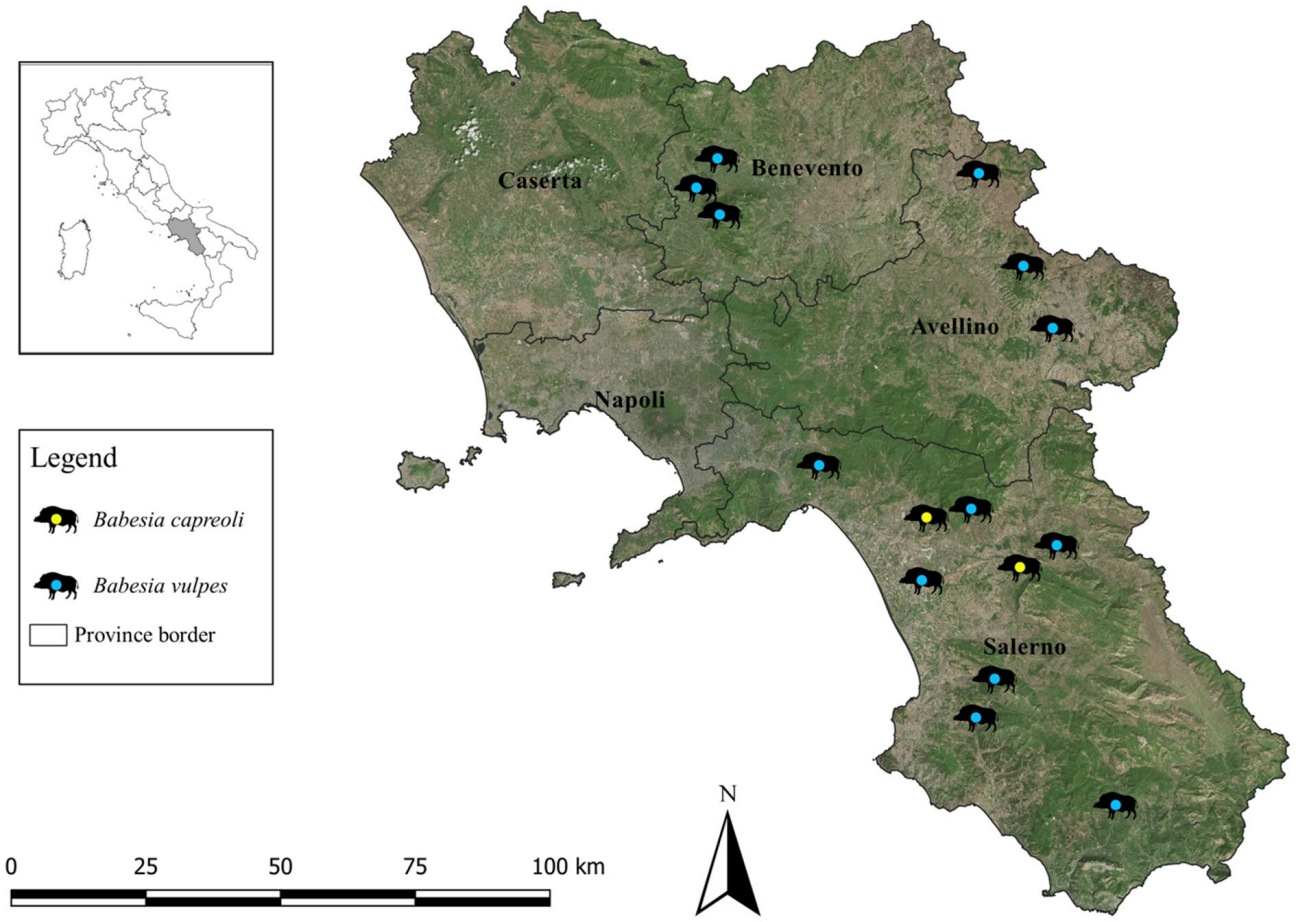
Distribution of wild boars (*n* = 15) positive to *Babesia vulpes* and *Babesia capreoli* by provinces (Avellino, Benevento, Caserta, Naples, Salerno) of the Campania region, southern Italy, 2016–2022.

A total number of 243 wild boar spleen samples from southern Italy between 2016 and 2022 were analyzed. Fifteen animals (i.e., 6.2, 95% CI: 3.4–9.9) tested positive to *Babesia* spp. DNA, 13 (i.e., 5.3, 95% CI: 3.1–8.9) and two (i.e., 0.8, 95% CI: 0.2–2.9) with *B. vulpes* and *B. capreoli*, respectively, using the combined semi-nested PCR/sequencing approach. The geographic distribution of *Babesia*-positive wild boars according to provincial borders of the study area is illustrated in Figure 1. Detailed data on prevalence, confidence intervals and statistical analyses are listed in Table 1. No statistically significant differences (i.e., *p* > 0.05) were found according to the boar’s age, sex, province and collection year. Three different *18S* rRNA partial sequences of *B. vulpes* were identified (sequence types ST1, ST2, ST3), with the most representative type being ST1 (60%) and a single sequence of *B. capreoli*. Compared to ST1, there were single nucleotide polymorphisms in ST2 (a T instead of C in position 220) and ST3 (a C instead of G in position 71). All sequences had 99–100% nucleotide identity with those available in GenBank. Sequences obtained in this study were deposited in GenBank under the following accession numbers: OQ520218 for *B. vulpes* ST1, OQ520219 for ST2, OQ520220 for ST3 and OQ520222 for *B. capreoli*.

**Table 1:** Wild boar spleen samples (*n* = 243) tested for *Babesia* spp. DNA in southern Italy, 2016–2022.

Variables	Pos/Tot^a^	%	95% CI^b^	Chi-squared; value of *p*	Odds ratio
**Age (years old)**					
Piglet (<1)	1/44	2.3	0.04–11.8		
Juvenile (1–2)	4/56	7.1	2.8–17.0		
Adult (>2)	10/143	7.0	3.8–12.4		
				*χ^2^ =* 1.4; *p* = 0.230	Not applicable
**Sex**					
Male	7/121	5.8	2.8–11.5		
Female	8/122	7.1	3.4–12.4		
				*χ^2^ =* 0.1; *p* = 0.800	0.88
**Province**					
Avellino	2/31	6.5	1.8–20.7		
Benevento	3/49	6.1	2.1–16.5		
Caserta	1/14	7.1	1.3–31.5		
Salerno	9/149	6.0	3.2–11.1		
				*χ^2^ =* 0.1; *p* = 0.998	Not applicable
**Year**					
2016	0/17	-	-		
2017	1/25	4.0	0.7–19.5		
2018	2/35	5.7	1.6–18.6		
2019	2/38	5.3	1.5–17.3		
2020	2/41	4.9	1.3–16.1		
2021	4/42	9.5	3.8–22.1		
2022	4/45	8.9	3.5–20.7		
				*χ^2^ =* 2.9; *p* = 0.820	Not applicable
Total	15/243	6.2	3.4–9.9		

a Pos/Tot: number of positive samples out of the total analyzed.

b 95% CI: 95% confidence interval.

## 4. Discussion

This study helps to fill the gap on *Babesia* spp. presence in wild boars, as well as suggesting cooperation of health stakeholders and trained persons (*citizen science* approach) as an effective tool for monitoring wildlife and related pathogens (26, 27).

To date, the only piroplasmid DNA in boars of Europe have been reported in a publication of *B. bigemina* in Italy (10), unspecified *Theileria* spp. in Italy and Portugal (8, 9) and *B. divergens* in the Czech Republic (7).

However, the moderate infection prevalence of *Babesia* spp. herein found in southern Italy (6.2%), and northern regions of the country (from 2.6% to 4.7%) (8, 10), suggests an involvement of boars in the sylvatic life cycle of the parasite. The absence of statistically significant difference in prevalence by boar’s age and sex in this study confirms that these variables do not influence the infection frequency, similar to findings of Zanet et al. (10). Again, the absence of significant differences in *Babesia* prevalence by province and collection year of samples suggests a stable circulation of infection in the study area.

Regarding *B. vulpes*, although the fox is the main reservoir in Europe (4, 28), the similar infection prevalence of this piroplasmid species in boars within this survey (13/243, 5.3%) and in foxes from the same study area (8/187, 4.3%) (29) indicates a potential involvement of this ungulate in pathogen maintenance. Despite roe deer being the most common host observed previously to be infected with *B. capreoli* (30, 31), its low prevalence (0.8%) in boars from this study should not exclude a role of these latter hosts in maintaining the pathogen considering the scant presence of other ungulate species in southern Italy, including roe deer (32). The potential pathogenic implications of *B. capreoli* infection in boars should be assessed in the future given that, although commonly asymptomatic in wildlife (6), cases of fatal babesiosis by this protozoan have been outlined in other wild ungulates, such as reindeer *Rangifer tarandus* (33) and Alpine chamois *Rupicapra rupicapra* (34, 35). Lastly, although not observed among boars in this study, the presence of suspected vectors of *B. vulpes* (i.e., *Ixodes hexagonus* and *Ixodes canisuga*) (36, 37) and *B. capreoli* (i.e., *Ixodes ricinus*) (38, 39) cannot be ruled out, considering that these tick species are commonly found on foxes (40) and hunting dogs (41) which live in sympatry with these ungulates. Indeed, due to the extensive time spent within sylvatic areas, hunting dogs show a higher prevalence of tick-borne pathogens compared to companion dogs (42). An example includes *B. vulpes* (43), capable to cause severe (4, 44) or fatal disease in dogs (45).

Despite the 18S rRNA gene is widely employed as a target for the molecular detection of piroplasmids (7, 14, 46, 47), the use of other genetic markers is recommended for species differentiation given the very high similarity of *Babesia* spp. sequences, such as *B. capreoli* and *B. divergens* which differ in just three positions (6, 30). Indeed, future studies on a larger sample size, including other wild ungulate species, and multiple genetic targets are needed to investigate the occurrence of piroplasmids in southern Italy.

The spread of wild boar populations may enhance the chance of transmission for emerging tickborne pathogens, including piroplasmids. More research is required to clarify the role of these ungulates in the maintenance of *B. vulpes* and *B. capreoli* in other epidemiological scenarios.

## Data availability statement

The datasets presented in this study can be found in online repositories. The names of the repository/repositories and accession number(s) can be found at: https://www.ncbi.nlm.nih.gov/nuccore; OQ520218, OQ520219, OQ520220, and OQ520222.

## Ethics statement

The animal study was approved by the project “Ricerca corrente” (grant number: IZSME RC 05/16) by the Italian Ministry of Health. Written informed consent was not required for this study in accordance with national legislation and institutional requirements.

## Author contributions

GS and VV conceptualized and designed the study. GS wrote the first draft of the manuscript. ND’A, CA, and HS wrote sections of the manuscript. AG, MGR, and FA performed molecular analyses. SR, SS, MO, and CDM were involved in sampling and database curation. GF and MGL managed project administration and resources. All authors contributed to the article and approved the submitted version.

## Funding

This work was supported by the project “Ricerca corrente” (grant number: IZSME RC 05/16) funded by the Italian Ministry of Health.

## Conflict of interest

The authors declare that the research was conducted in the absence of any commercial or financial relationships that could be construed as a potential conflict of interest.

## Publisher’s note

All claims expressed in this article are solely those of the authors and do not necessarily represent those of their affiliated organizations, or those of the publisher, the editors and the reviewers. Any product that may be evaluated in this article, or claim that may be made by its manufacturer, is not guaranteed or endorsed by the publisher.
